# Effects of lignin modification on wheat straw cell wall deconstruction by *Phanerochaete chrysosporium*

**DOI:** 10.1186/s13068-014-0161-3

**Published:** 2014-11-29

**Authors:** Jijiao Zeng, Deepak Singh, Difeng Gao, Shulin Chen

**Affiliations:** Department of Biological Systems Engineering, Bioprocessing and Bioproduct Engineering Laboratory (BBEL), Washington State University, L.J. Smith 213, Pullman, Washington 99163 USA

**Keywords:** NMR, Lignin degradation, White rot fungi, Lignin-carbohydrate complex, Cellulase absorption, Solid-state NMR, Inter-unit linkages, Milled wood lignin, Cellulase enzyme lignin

## Abstract

**Background:**

A key focus in sustainable biofuel research is to develop cost-effective and energy-saving approaches to increase saccharification of lignocellulosic biomass. Numerous efforts have been made to identify critical issues in cellulose hydrolysis. Aerobic fungal species are an integral part of the carbon cycle, equip the hydrolytic enzyme consortium, and provide a gateway for understanding the systematic degradation of lignin, hemicelluloses, and cellulose. This study attempts to reveal the complex biological degradation process of lignocellulosic biomass by *Phanerochaete chrysosporium* in order to provide new knowledge for the development of energy-efficient biorefineries.

**Results:**

In this study, we evaluated the performance of a fungal biodegradation model, *Phanerochaete chrysosporium,* in wheat straw through comprehensive analysis. We isolated milled straw lignin and cellulase enzyme-treated lignin from fungal-spent wheat straw to determine structural integrity and cellulase absorption isotherms. The results indicated that *P. chrysosporium* increased the total lignin content in residual biomass and also increased the cellulase adsorption kinetics in the resulting lignin. The binding strength increased from 117.4 mL/g to 208.7 mL/g in milled wood lignin and from 65.3 mL/g to 102.4 mL/g in cellulase enzyme lignin. A detailed structural dissection showed a reduction in the syringyl lignin/guaiacyl lignin ratio and the hydroxycinnamate/lignin ratio as predominant changes in fungi-spent lignin by heteronuclear single quantum coherence spectroscopy.

**Conclusion:**

*P. chrysosporium* shows a preference for degradation of phenolic terminals without significantly destroying other lignin components to unzip carbohydrate polymers. This is an important step in fungal growth on wheat straw. The phenolics presumably locate at the terminal region of the lignin moiety and/or link with hemicellulose to form the lignin-carbohydrate complex. Findings may inform the development of a biomass hydrolytic enzyme combination to enhance lignocellulosic biomass hydrolysis and modify the targets in plant cell walls.

**Electronic supplementary material:**

The online version of this article (doi:10.1186/s13068-014-0161-3) contains supplementary material, which is available to authorized users.

## Background

Lignocellulosic biomass consists of *β*-(1,4)-linked glucose polymer cellulose, hemicellulose polysaccharides of varying composition, and lignin. To increase the sugar yield from biomass, pretreatment technologies primarily lead to three main physicochemical alterations: 1) cleavage of lignin-carbohydrate bonds and removal of hemicellulose, 2) lignin modification and degradation, and 3) decrystallization of cellulose [1]. Two of these three relate to lignin and its derivatives. Lignin fills the spaces in the cell wall between cellulose, hemicellulose, and pectin components, especially in tracheid, sclereid, and xylem cells [2]. It also links hemicellulose [1,3] depositing on the cellulose surface, thus constituting an obstruction for effective hydrolysis.

Structurally, lignin is a polymer of heterogeneous phenylpropanoid units in vascular plants that is built randomly by oxidative coupling between hydroxyphenyl (H), guaiacyl (G), and syringyl (S), which are derived from three corresponding monolignols: *p*-coumaryl alcohol (*p*CoumA), coniferyl alcohol (ConA), and sinapyl alcohol (SinA). This leads to the most chemically resistant structures due to aromaticity and a diverse set of C-C and C-O cross-links [4]. Moreover, due to variations in the coupling process and the distribution of basic units in plant tissue, the structural properties are dictated by the variety of chemically distinct linkages [5,6]. However, their roles in the enzymatic hydrolysis of cellulose are not fully understood. Lignin has been found to spontaneously absorb cellulase [7,8]. The microfibrillar architecture analysis of cell walls suggests that digestion minimizes the lignin barrier and maintains the native polysaccharide architecture [9]. On the other hand, the correlation of lignin content to cellulose hydrolysis is weak in terms of functionality and composition [10]. In fact, high levels of delignification without a significant increase in cellulose accessibility do not result in the expected digestibility [11].

Biodegradation systems such as *Phanerochaete chrysosporium* form an enormous source of lignocellulolytic enzymatic complexes [12]. These naturally provide novel resources for the delignification of bioenergy crops and other sources of lignocellulosic biomass [13,14]. During delignification, three predominant reactions occur: 1) side chain oxidation (C_*α*_-C_*β*_ cleavage), 2) ring hydroxylation, and 3) demethylation [15]. The current model for lignin breakdown is derived from studies of fungal metalloenzymes and based on oxidative combustion of lignin through various radical-mediated paths. This process ultimately generates phenoxyl and phenyl radicals on the substrate, followed by depolymerization [16,17]. Lignin peroxidase (LiP) executes the H_2_O_2_-dependent C_*α*_-C_*β*_ cleavage of lignin, based on the reports on LiP-dependent model compound degradation, which is subsequently shown to catalyze the partial depolymerization of methylated lignin *in vitro* [18,19]. Nonphenolic syringyl and biphenyl model compounds are oxidized by LiP. In contrast, manganese peroxidase (MnP) is not effective in countering nonphenolic lignin degradation. However, biomimetic oxidation of lignin model compounds by Mn^3+^ suggest that it may play a role in oxidizing both phenolic and nonphenolic residues of lignin [20] via a lipid peroxidation reaction [21] or in the presence of cellobiose dehydrogenases (CDH) [22].

The role of lignin biodegradation during the fungal utilization of lignocellulosic biomass is still not fully understood [23,24]. In fact, there is little evidence to support the concept of lignin being used as a carbon source or nutrient to increase growth. Biodelignification is generally thought to either increase cellulose accessibility, retain cellulase activity, or both. Previous studies suggest that clarification of a detailed skeleton of plant cell walls during fungal biodegradation can enhance biomass conversion technology [23]. Great efforts have been made in investigating lignin biodegradation reactions [15]; however, the role of lignin in subsequent cellulose digestion in biological systems is still ambiguous.

This study attempts to evaluate the key steps of lignin biodegradation on wheat straw and it explores new biological strategies for lignin degradation and its potential for biomass hydrolysis technology. The findings provide detailed structural data on lignin from fungal-spent wheat straw through comprehensive NMR analysis. This study also explores the adsorption isotherms of cellulases for lignin in order to evaluate the influence of lignin adsorption capacity on cellulose hydrolysis.

## Results and discussion

**Figure 1:**
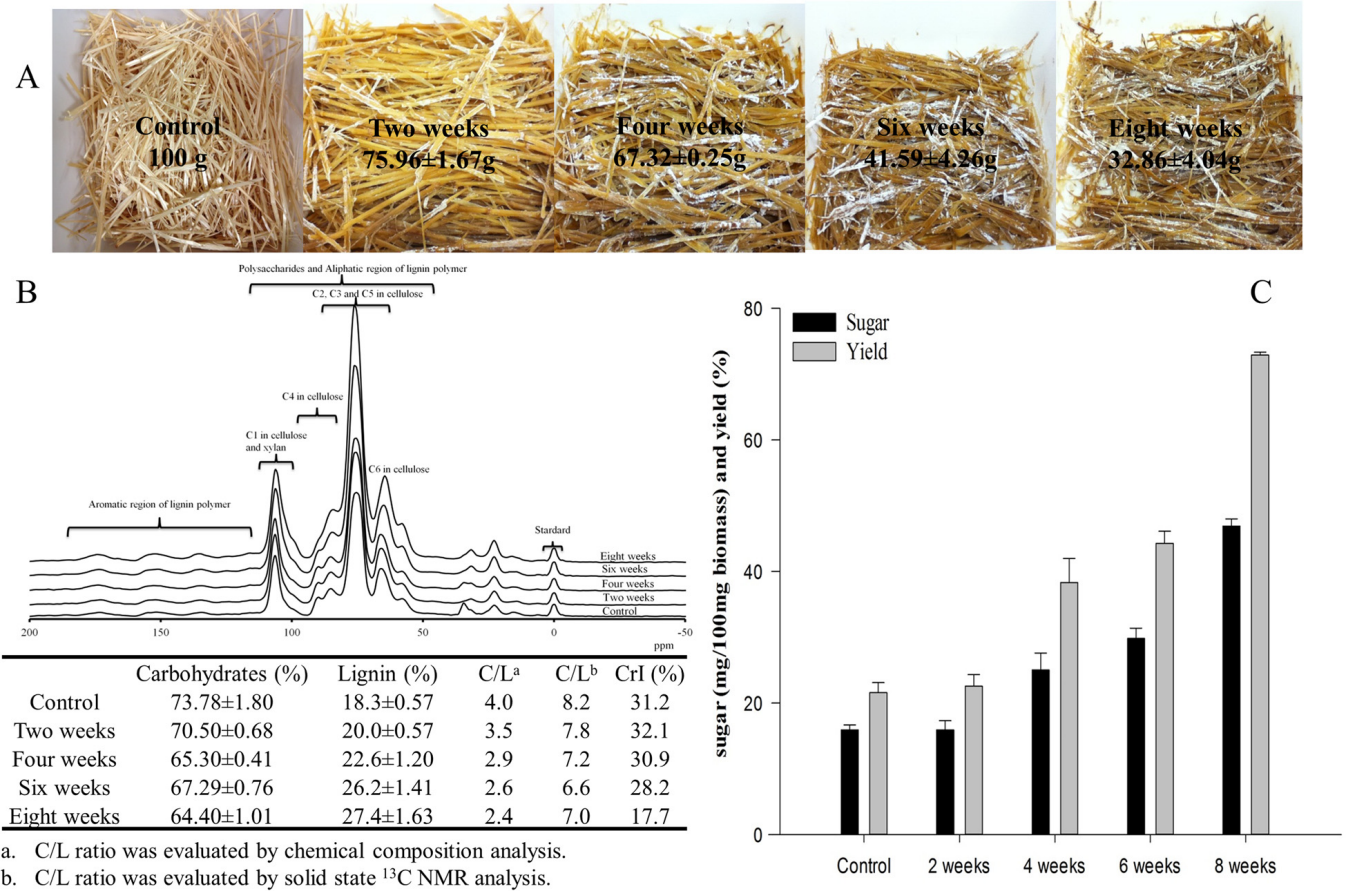
**Degradation of wheat straw by** ***P. chrysosporium*** **with different incubation periods (A), chemical compositional structural analysis (B), and enzymatic hydrolysis (C).** Sterilized wheat straw samples (100 g) were treated in plastic containers inside a low temperature 815 incubator (Precision Scientific) **(A)**. The containers were covered with eight-layer gauze pads in order to allow air exchange. Every 48 to 72 hours, 50 mL of water was evenly sprayed on the surface of the wheat straw while manually rotating the samples. At the end of the incubation periods, each group was collected individually, washed with water, dried, and milled to a fine powder (44 to 74 um).

The growth of *P. chrysosporium* on wheat straw led to a significant weight reduction in biomass, which was dependent on the treatment time (Figure 1A). After eight weeks of fungal treatment, the wheat straw mass decreased dramatically to 32.86 ± 4.04 g from 100 g original dry weight (about 70% less than the initial weight of the biomass). The first two weeks of treatment involved the most dramatic weight loss of biomass at about 20% (Additional file 1: Table S1). Compositional analysis revealed the simultaneous degradation of lignin and carbohydrates. However, the relative reduction of carbohydrate/lignin (C/L) ratio was also recorded in the treated samples. The C/L ratio decreased from 4.0 to 2.4 within eight weeks of incubation time (Figure 1B), indicating a substantial utilization of carbohydrates compared to lignin. This diminished C/L ratio was confirmed by solid-state ^13^C NMR analysis.

The peak area from 93 to 82 ppm was assigned to crystalline and amorphous signals for C4 of the cellulose polymer [25] (Figure 1B). Crystallinity estimates for the biomass (CrI) ranged from 32.1 to 17.7% for two to eight weeks of incubation (Figure 1B). The increased efficiency of enzymatic hydrolysis of the fungal-spent straw is in accordance with the reduced crystallinity. The total sugar release was three to four times higher in the wheat straw treated for eight weeks compared to the control (Figure 1C). The cellobiose oxidase and cellobiose quinone oxidoreductase derived from *P. chrysosporium* were found to strongly bind with crystalline region of cellulose [26,27], enhancing the crystalline cellulose degradation by cellulases [27]. But due to the blurred boundary between the crystalline and amorphous regions (Figure 1B), the CrI of the eight-week sample might be underestimated.

*P. chrysosporium* has a strong ability to produce hydrolytic enzymes that dissociate numerous interlocks in biomass substructures. These include various glycoside hydrolase (GH) family proteins, such as cellobiohydrolase (EC 3.2.1.4), endoglucanase (EC 3.2.1.4), cellobiose dehydrogenase (EC 1.1.99.18), *β*-glucosidase (EC 3.2.1.21), xylanase (EC 3.2.1.8), polygalacturonase (EC 3.2.1.15), xylan esterase (EC 3.2.1.72), arabinase (EC 3.2.1.99), and mannanases (EC 3.2.1.78), as well as lignin degradation enzymes, including lignin peroxidase (LiP) (EC 1.11.1.14), manganese peroxidase (MnP) (EC 1.11.1.13), copper radical oxidases (EC 3.2.1.4), glyoxal oxidase (E.C 1.1.3.-), and quinone oxidoreductase (EC 1.6.99.21) [12,24,28,29]. During biological degradation of the wheat straw, our findings show that over 50% of the carbohydrate and lignin was digested within eight weeks (Additional file 1: Table S1). However, the changes in C/L ratios (Figure 1B) suggest that lignin degradation gradually slowed down compared to consumption of total cellulose and hemicellulose. In contrast, high lignin content did not affect the saccharification efficiency (Figure 1C).

Studies show that cellulose accessibility [11,30] and lignin composition/characteristics [10] strongly influence enzymatic hydrolysis. Elevated enzymatic hydrolysis for the fungal-spent biomass can be partially explained by a decrease in CrI (Figure 1B). However, it is possible that biologically degraded lignin may lower absorption effects on cellulase and/or inhibition to cellulases because the active functional groups are saturated or otherwise unavailable during fungal growth in biomass.

**Figure 2:**
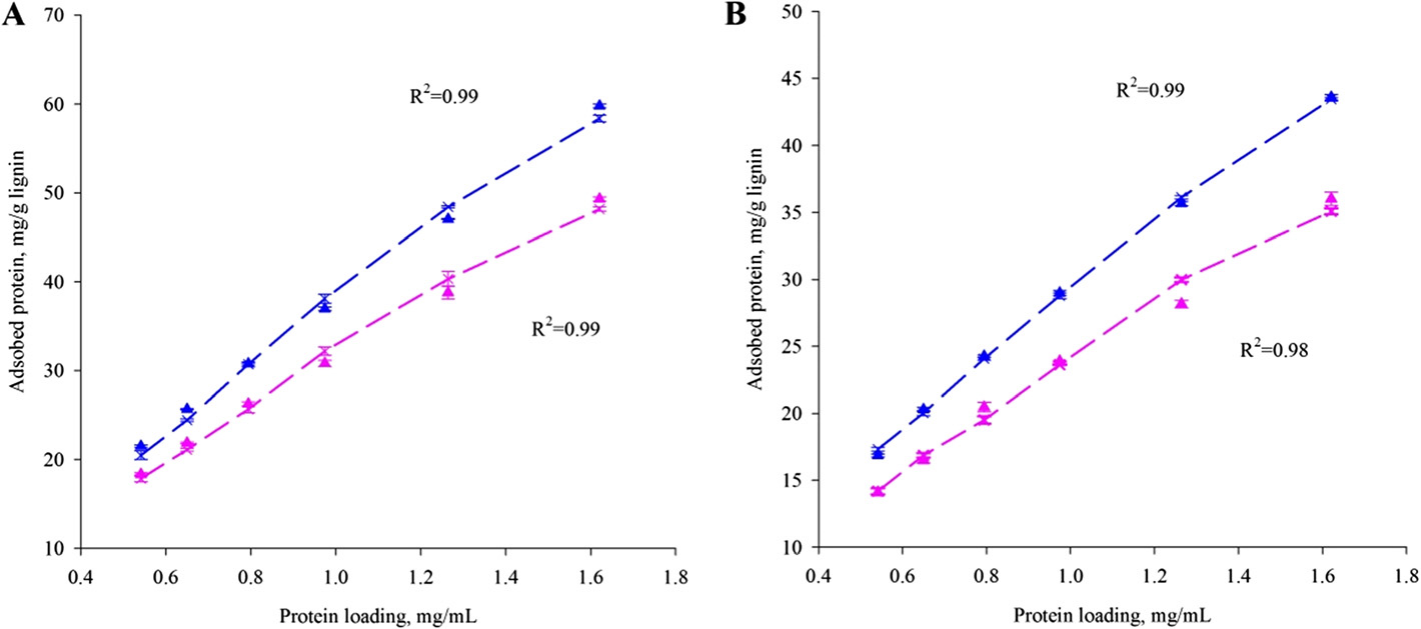
**Cellulase adsorption on MSL (A) and CEL (B) at 4°C.** Adsorbed cellulase on control lignin (pink) and eight weeks treated lignin (blue) was determined by subtraction of protein in supernatant from total loaded protein. Experimental data (triangles), data predicted by Langmuir equation (crosses with dashed lines). Error bars represent standard deviation.

To test this hypothesis, we isolated milled straw lignin (MSL) and cellulase enzyme lignin (CEL) and used them to represent the possible influence of chemical properties on cellulase adsorption. As shown in Figure 2, the adsorption data followed the Langmuir isotherm well (R^2^ > 0.95), estimated with nonlinear regression using Polymath. This verified that biologically modified wheat straw lignin absorbs more cellulase than native lignin. MSL showed higher maximum adsorption abilities than CEL (Table 1). Treated samples of both types of lignin had higher maximum adsorption capacities than untreated lignin, as well as higher affinity. The range of adsorption parameters (maximum adsorption capacity and affinity) agreed with previous studies that used chemical pretreatments, such as ammonia fiber expansion (AFEX), dilute acid, organosolv, and SO_2_-steam [7,31,32] (Table 1). MSL showed a higher maximum adsorption capacity than CEL, presumably because MSL contains lower amounts of carbohydrate residues. This results in higher exposure of lignin units to cellulase (Additional file 2: Table S2). However, against our expectations, lignin that is biologically degraded and/or modified by *P. chrysosporium* could capture more cellulase in both MSL and CEL.

**Table 1:** **Parameters of cellulase adsorption on MSL and CEL isolated from biological treated and untreated wheat straw**

**Lignin isolation**	**°C**	**Biomass**	**Pretreatment**	**σ (mg/g lignin)**	**A (L/g)**	**S = σ × A (mL/g)**	**Reference**
EnzL	4	Poplar	AFEX	56.8	2.14	121.6	Kumar, R and C.E. Wyman, 2009 [7]
EnzL	4	Corn stover	DA	53	0.68	36.0	Kumar, R and C.E. Wyman 2009 [7]
CEL	50	Douglas fir	SO_2_-steam	79.3	1.86	147.5	Nakagame, S., *et al.*, 2011a [31]
CEL	50	Lodgepole pine	organosolv	34.9	1.43	49.9	Nakagame, S., *et al.*, 2011b [32]
MSL	4	Wheat straw	Untreated	136.2	0.86	117.4	In this study
MSL	4	Wheat straw	*P. chrysosporium*	171.1	1.22	208.7	In this study
CEL	4	Wheat straw	Untreated	86.9	0.75	65.3	In this study
CEL	4	Wheat straw	*P. chrysosporium*	100.4	1.02	102.4	In this study

The adsorption capacity (σ), affinity constant (A), and strength of binding (S) were evaluated by fitting Langmuir isotherm model. EnzL: enzyme lignin; AFEX: ammonia fiber expansion; DA: dilute acid.

Hydrophobicity (lignin part) or hydrophilicity (carbohydrate part) of lignin-carbohydrate complexes (LCCs) may produce nonproductive (retarding saccharification) as well as productive (facilitating saccharification) binding of enzymes, respectively [32]. Hydrophobic interaction is considered to be the major interaction between lignin and cellulases [31,33,34,35]. It is also possible that some GH homologs with a cellulose-binding domain (CBD) may bind to the surface of the MSL/CEL’s LCC, causing a higher cellulase adsorption ability. The CBDs of families I, II, and III have a carbohydrate-binding cleft where a set of aromatic residues and a group of polar residues occur [36]. However, cellulose hydrolysis results showed a higher sugar yield for modified lignin (Figure 2 and Table 1) when cellulases are added in wheat straw saccharification (Figure 1). Therefore, this effect cannot be explained by reducing the unproductive binding of cellulases to lignin.

**Figure 3:**
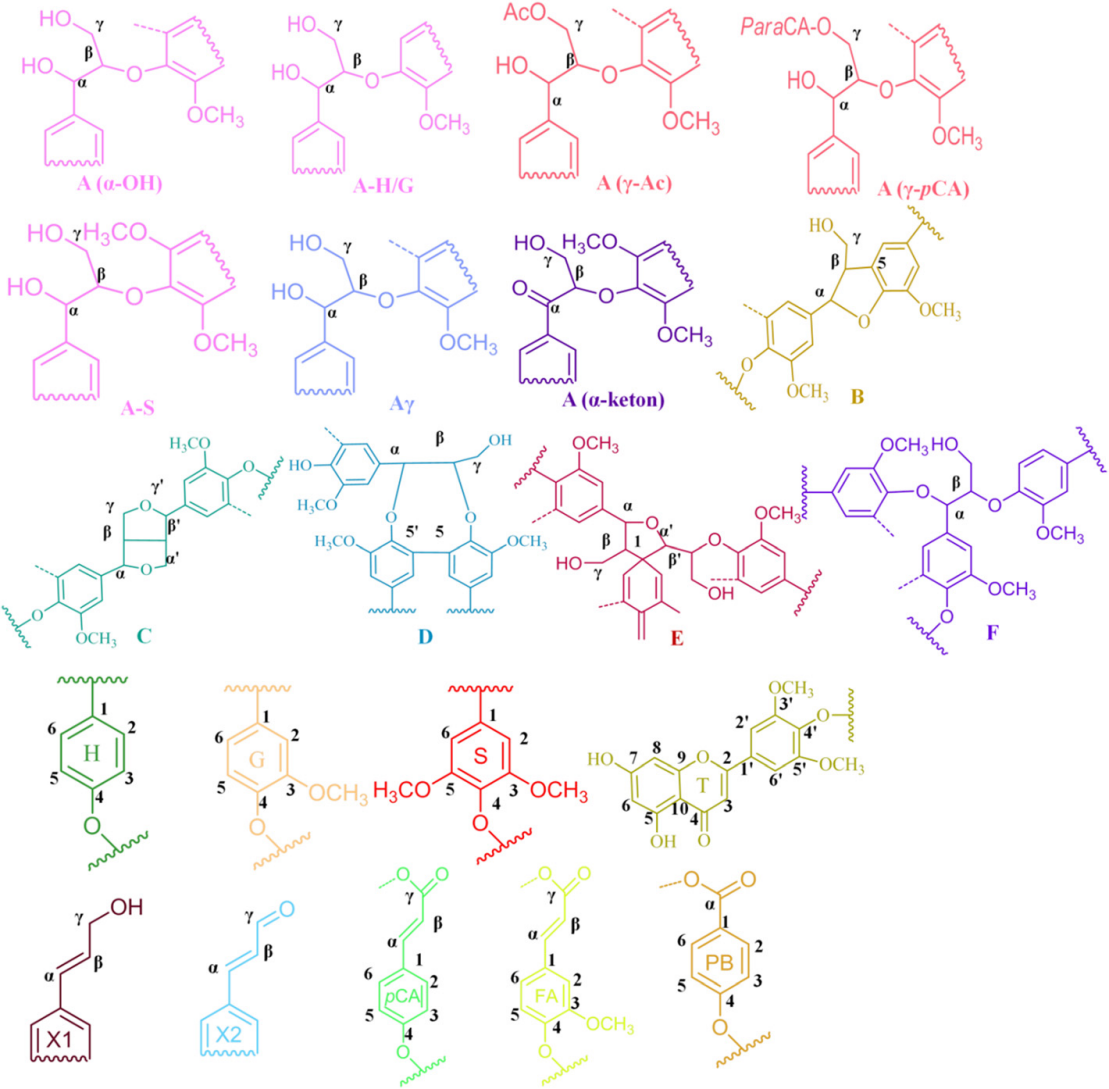
**Main lignin units and inter-unit linkages in wheat straw.** A(α-OH): α-OH in *β*-O-4 linkage; A-H/G: coupled with H or G unit in *β*-O-4 linkage; A-S: coupled with S unit in *β*-O-4 linkage; A(γ-Ac): acylation by acetate in γ-OH; A(γ-*p*CA): acylation by *p*CA in γ-OH; Aγ: γ-OH in *β*-O-4 linkage; B: phenylcoumaran (*β*-5); C: pino/resinol (*β*-β); D: dibenzodioxocins (5-5′/4-O-*β*'); E:Spirodienone (*β-*1);F: *α,β*-diaryl ethers; H: *p*-hydroxyphenyl units; G: guaiacyl units; S: syringyl units; T: tricin units; X1: cinnamyl alcohol; X2: cinnamyl aldehyde; *p*CA: *p*-coumarate; FA: ferulate; PB: hydroxybenzoate.

**Figure 4:**
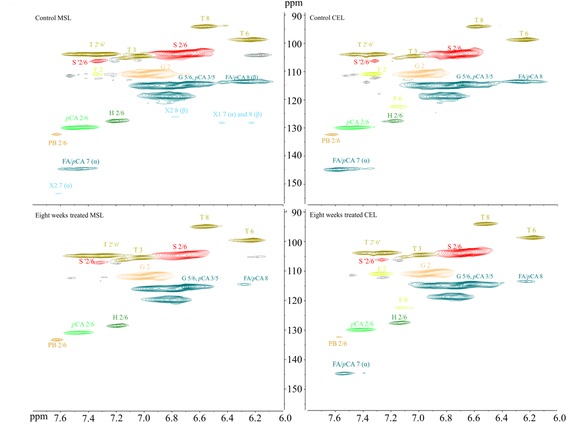
**The aromatic region of MSL and CEL isolated from eight weeks, treated and untreated, wheat straw in HSQC spectra.** H: *p*-hydroxyphenyl units; G: guaiacyl units; S: syringyl units; T: tricin units; X1: cinnamyl alcohol; X2: cinnamyl aldehyde; *p*CA: *p*-coumarate; FA: ferulate; PB: hydroxybenzoate.

**Figure 5:**
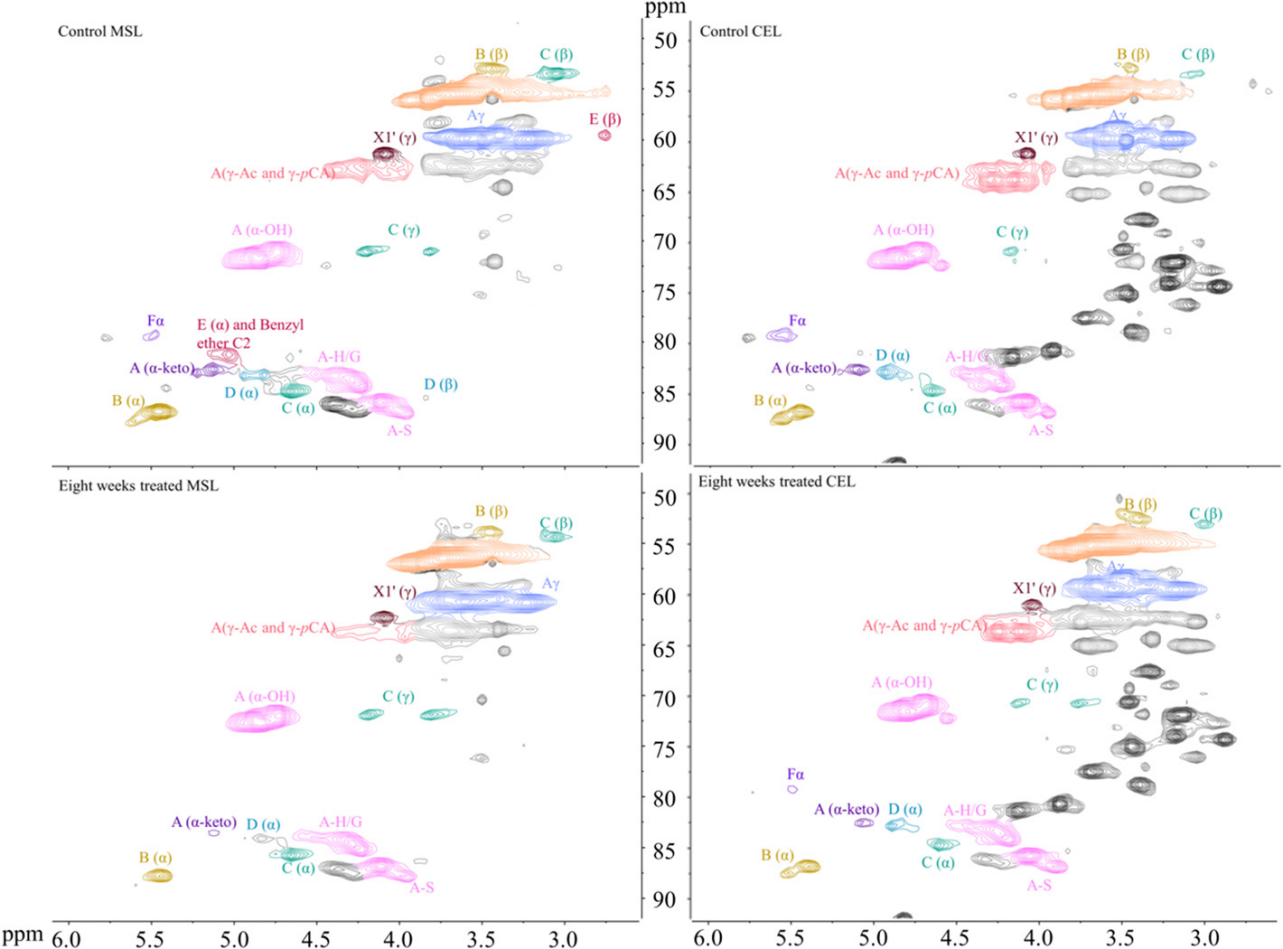
**The aliphatic region of MSL and CEL isolated from eight weeks, treated and untreated, wheat straw in HSQC spectra.** A(α-OH): α-OH in *β*-O-4 linkage; A-H/G: coupled with H or G unit in *β*-O-4 linkage; A-S: coupled with S unit in *β*-O-4 linkage; A(γ-Ac): acylation by acetate in γ-OH; A(γ-*p*CA): acylation by *p*CA in γ-OH; Aγ: γ-OH in *β*-O-4 linkage; B: phenylcoumaran (*β*-5); C: pino/resinol (*β*-β); D: dibenzodioxocins (5-5′/4-O-*β*'); F: *α,β*-diaryl ethers.

To explore the potential role of lignin degradation on saccharification, we performed a structural analysis of lignin with heteronuclear single quantum coherence spectroscopy (HSQC) NMR. The spectra were compared for untreated milled straw lignin (MSL) and cellulase enzyme lignin (CEL), with major structures in aromatic (δ_C_/δ_H_ 160-102/8.0-6.0 ppm) and aliphatic regions (δ_C_/δ_H_ 90-50/6-3 ppm). The major structures and inter-unit linkages are listed in Figure 3. Previous studies detail the chemical shifts and assignments of various lignin moieties from wheat straw [36]. In the aromatic regions of HSQC spectra, tricin (T), guaiacyl lignin (G), syringyl lignin (S), *p*-coumarate (*p*CA), and ferulate (FA) were found to form the backbone of wheat straw lignin (Figure 4). Differences were mainly observed in terms of signal reduction corresponding to α/β correlations from ferulate and *p*CA, as well as cinnamyl alcohol (X1) and cinnamyl aldehyde (X2). This suggests a queue of lignin degradation patterns by this fungus on wheat straw. The aliphatic regions of HSQC spectra of MSL and CEL show comparable signals from arylglycerol-β-aryl ether (*β*-O-4), pino/resinol (*β-β'*), and dibenzodioxocin (5-5*′/*4-O-*β'*) linkages. However, weaker correlations of γ-ending groups, γ-acylation groups, phenylcoumaran (*β*-5), and α,β-diaryl ether (*α-*O*-*4*/β-*O-4) were detected (Figure 5). Polysaccharide signals were also intensified in CEL, possibly due to exposure of LCCs by additional cellulase treatment of the lignin residues, which exhibited more *β*-D-xylan and arabinose [38].

Quantification of the lignin units and inter-unit linkages from isolated MSL and CEL are summarized in Table 2. Interestingly, *P. chrysosporium* led to relatively low values in S/G ratios, with increased T units from 8.5 to 12 units in MSL, and from 5.6 to 7.3 units in CEL, respectively. The lower S/G ratios in fungal biodegradation resulted in a greater cellulose accessibility. Higher S/G ratios in the deposited lignin during the hydrothermal pretreatment are reported to inhibit cellulose hydrolysis [39]. Moreover, reduction of hydroxybenzoate (PB), *p*CA, and FA units subsequently lower cinnamate/lignin ratios. The *β*-O-4, *β*-5, and *β-β'* inter-unit linkages of biologically treated and untreated lignin occupy over 90% of side chains with the exception of the D and F units in the aliphatic region. A comparison of isolated MSL and CEL further reveals that *P. chrysosporium* degrades the B and F units compared to the A, C, and D units. Special attention was paid to γ-ending groups, as they strongly relate with hydroxycinnamates. Consistent with results for the aromatic region (Figure 4 and Table 2), γ-OH in the X1 structure decreased from 2.0 to 1.2 units in MSL, and from 1.5 to 0.9 units in CEL. γ acylation decreased from 5.4 to 2.0 units in MSL, and from 15.6 to 9.8 units in CEL. We further analyzed CEL lignin that was isolated from wheat straw treated for two, four, and six weeks with quantitative NMR (Additional file 3: Figure S1 and Additional file 4: Figure S2). Quantitative analysis revealed similar trends for the modification of lignin (Table 3).

**Table 2:** **Quantification of aromatic moieties (lignin, hydroxycinnamates, inter-unit bondages) by combination analysis of** ^**13**^ **C and HSQC NMR spectra of wheat straw isolated MSL and CEL**

**Aromatic moieties**	**σ**_**C**_ **/σ**_**H**_ **(ppm) and assignments**	**Control MSL**	**Control CEL**	**Eight weeks MSL**	**Eight weeks CEL**
Lignin aromatic units					
Syringyl (S)	103.9/6.7 (S2/6),	25.7	27.3	24.1	24.5
	106.4/7.4 (S'2/6 with α oxidization)				
Guaiacyl (G)	110.8/6.97 (G2)	41.2	39.3	41.6	39.8
	115.0/6.94 (G5),118.9/6.81 (G6)				
*p*-hydroxyphenyl (H)	127.7/7.21 (H2/6)	1.6	1.4	2.3	1.8
S/G ratio		0.63	0.69	0.58	0.61
Hydroxycinnamates					
*p-*benzoate (PB)	132.4/7.63 (PB2/6)	0.5	1.2	1.3	0.2
*p-*coumarate (*p*CA)	130.0/7.47 (*p*CA2/6),115.5/6.79 (*p*CA3/5)	5.4	5.5	3.0	3.1
	144.74/7.47				
Ferulate (FA)	110.9/7.32 (FA2), 122.5/7.15 (FA6)	0.6	6.4	0.4	4.5
*p*-coumarate/ferulate		9	0.86	7	0.68
Cinnamates/lignin and tricin		0.09	0.18	0.06	0.11
Lignin-ending group					
Cinnamyl aldehyde (X2)	153.5/7.62 (X2α), 126.2/6.78 (X2β)	0.1	ND	ND	ND
Cinnamyl alcohol (X1)	128.34/6.44 (X1α), 128.35/6.22 (X1β)	0.6	ND	ND	ND
Lignan					
Tricin (T)	104.04/7.30 (T 2′/6'), 104.65/7.03 (T3)	8.5	5.6	12	6.6
	94.1/6.56 (T8), 98.8/6.22 (T6)				
Inter-unit bondages					
α-OH/β-O-4	71.1/4.74 (Aα-G), 71.8/4.8 (Aα-S)	28.3	33.8	31.7	36.5
α-keto/β-O-4	82.7/5.1 (Aα-keto)	1.5	2.4	0.4	1.1
Total β-O-4		29.8	36.2	32.1	37.6
Phenylcoumaran (*β-5*)	85.9/5.5 (Bα), 53.0/3.4 (Bβ)	5.4	5.7	3.0	4.1
Pino/resinol (*β-β'*)	84.8/4.7 (Cα), 53.5/3.1 (Cβ),	1.3	0.9	1.4	1.2
Dibenzodioxocin (D)	83.4/4.9 (Dα), 85.5/3.8 (Dβ)	0.3	2.0	0.7	2.5
Spirodienone (SD)	81.4/5.0 (SDα), 59.5/2.8 (SDβ)	0.4	ND	ND	ND
α,β-diaryl ether (F)	79.6/5.5 (Fα)	0.5	2.1	0.4	1.3
γ-ending groups					
γ-OH in X1 structure	62-58/3.8-3.0	2.0	1.5	1.2	0.9
Acylation on γ position	60.8-58.8/3.8-3.35	5.4	15.6	2.0	10.1

**Table 3:** **Quantification of aromatic moieties (lignin, hydroxycinnamates, inter-unit bondages) by combination analysis of** ^**13**^ **C and HSQC NMR spectra of wheat straw isolated CEL**

**Aromatic moieties**	**σ**_**C**_ **/σ**_**H**_ **(ppm) and assignments**	**Control CEL**	**Two weeks CEL**	**Four weeks CEL**	**Six weeks CEL**	**Eight weeks CEL**
Lignin aromatic units						
Syringyl (S)	103.9/6.7 (S2/6),	27.3	20.5	19.4	21.3	24.5
	106.4/7.4 (S'2/6 with α oxidization)					
Guaiacyl (G)	110.8/6.97 (G2)	39.3	40.8	37.2	40.9	39.8
	115.0/6.94 (G5),118.9/6.81 (G6)					
*p*-hydroxyphenyl (H)	127.7/7.21 (H2/6)	1.4	2.2	2.4	2.3	1.8
S/G ratio		0.69	0.50	0.52	0.52	0.61
Hydroxycinnamates						
*p-*benzonate (PB)	132.4/7.63 (PB2/6)	1.2	ND	ND	ND	0.2
*p-*coumarate (*p*CA)	130.0/7.47 (*p*CA2/6),115.5/6.79 (*p*CA3/5)	5.5	2.5	2.7	3.3	3.1
Ferulate (FA)	110.9/7.32 (FA2), 122.5/7.15 (FA6)	6.4	3.9	4.6	5.4	4.5
*p*-coumarate/ferulate		0.86	0.65	0.58	0.60	0.68
Cinnamates/lignin and tricin		0.18	0.09	0.10	0.12	0.11
Lignan						
Tricin (T)	104.04/7.30 (T 2′/6'), 104.65/7.03 (T3)	5.6	10	12	9.0	6.6
	94.1/6.56 (T8), 98.8/6.22 (T6)					
Inter-unit bondages						
α-OH/β-O-4	71.1/4.74 (Aα-G), 71.8/4.8 (Aα-S)	33.8	33.9	28.1	33.9	36.5
α-keto/β-O-4	82.7/5.1 (Aα-keto)	2.4	0.8	0.6	0.8	1.1
Total β-O-4		36.5	34.7	28.7	34.7	37.6
Phenylcoumaran (*β*-5)	85.9/5.5 (Bα), 53.0/3.4 (Bβ)	5.7	4.2	3.3	5.2	4.2
Pino/resinol (*β-β'*)	84.8/4.7 (Cα), 53.5/3.1 (Cβ),	0.9	1.2	1.6	1.5	1.2
Dibenzodioxocin (D)	83.4/4.9 (Dα), 85.5/3.8 (Dβ)	2.0	3.8	2.7	4.0	2.5
Spirodienone (SD)	81.4/5.0 (SDα), 59.5/2.8 (SDβ)	ND	ND	ND	ND	ND
α,β-diaryl ether (F)	79.6/5.5 (Fα)	2.1	ND	ND	ND	1.3
γ-ending groups						
γ-OH in X1 structure	62-58/3.8-3.0	1.5	0.9	0.8	1.0	0.9
Acylation on γ position	60.8-58.8/3.8-3.35	15.6	11.1	13.1	11.8	10.1

Clearly, the relative decrease in S units along with the relative increase in G units caused an overall decrease in S/G ratios. Two key points explain the “fragility” of the S unit [40] over the G unit: i) S units have less redox potential (are more easily oxidized) than G and H units; and ii), due to the non-availability at the 5 position (taken by the methoxy group), S units can easily form linear structures by *β-*O-4 [41] rather than *β-5* and 5-5 structures. In addition, the spatial distribution of the syringyl unit was determined by Zhou *et al.* (2011) [42], who found that it showed a preferential localization in the fiber cell wall and secondary cell wall of a plant cell. Polysaccharides are also deposited in the secondary cell wall [43]. It is possible that fungi “choosing” to degrade the S units may be ascribed to real-life needs: 1) S units are relatively delicate; (2) removing S units may increase the accessibility of cellulose to cellulases.

Changes in the S/G ratio also contribute to fluctuation in T units. The incorporation of tricin units into herbaceous lignin [44] provides new insight into understanding the structural integrity of lignin. Our findings show that the lignin content of wheat straw strongly relies on the S/G ratio, at up to 8 units per 100 Ar [37]. Tricin is involved in linking to G units via *β-*O-4 [44], which can misdirect the S/G ratio by other analytical approaches and lead to overestimation of S units content at the end [23,45]. Gradually increasing the tricin content (Table 1 and Table 2) in *P. chrysosporium*-spent wheat straw suggested that tricin was not primarily involved in the biodegradation of lignin.

The decrease of hydroxycinnamates (FA, PB, and *p*CA) (Table 2 and Table 3) is a typical fingerprint in biologically treated wheat straw, which contributes to free phenolic OH and carboxylic/carbonyl groups in herbaceous lignin. Improvement of lignocellulosic digestibility by white rot fungi is consistent with degradation of *p*CA and FA [46]. FA is primarily esterified to arabinosyl residues of arabinoxylan chains, and feruloylated arabinoxylans are later cross-linked to G units of lignins via ether bonds. Since FA is involved in ester linkages and phenol coupling reactions, it can covalently attach polysaccharide with lignin. The resultant product is a ferulate-polysaccharide-lignin complex that is bonded through ester-ether linkages [47]. Thus, FA deposition may not only lead to cell wall cross-linking during plant growth and development, but may also regulate the non-random pattern of lignin formation within the wall [48].

The elimination of FA/*p*CA α/β’ correlations (Figure 4 and Additional file 3: Figure S1) and the decrease of FA (Table 2 and Table 3) and arabinose content (Additional file 1: Table S1) confirm the cleavage of the labile ester side chain of ferulate during biodegradation. Studies show that γ-OH in wheat straw lignin is widely acylated by *p*CA and acetate [43], while *p*CA also can couple with the α position of lignin by ether linkage [49]. S units are enzymatically pre-acylated with *p*CA [4] before incorporation into the lignin complex via *β*-5 or *β-β'* rather than *β*-ether in wheat straw [43]. The deconstruction of *β*-5 and α,β ether linkages (Figure 5, Table 2 and Table 3) suggests the potential incorporation pathway between hydroxycinnamate and lignin units in wheat straw. In addition, the degradation of hydroxycinnamates is possibly ascribed to the ligninase system of *P. chrysosporium*. The *p*CA is mostly presented as a free phenolic compound in the entire lignin complex, favored by MnP through α-β cleavage reactions (white rot fungi degradation style) [13,15,50]. The primary targets of hydroxycinnamates by *P. chrysosporium* during biodegradation of wheat straw lignin offer new opportunities in lignin biosynthesis to improve the biomass saccharification process.

Recent studies reveal the adverse effect of phenolics on cellulase activity [51]. Improved digestibility of biomass may also be due to the breakdown of the lignin-carbohydrate complex (LCC) [4]. LCCs have been compared to the hemicelluloses, which may protect the cell wall from enzyme attack, assuming the low LCCs during biological pretreatment. This facilitates the accessibility of cellulose due to inherently bigger pores that the enzymes can penetrate (about 4 to 13 nm). Arabinoxylan acylated FA plays a very important role in bridging lignin and hemicellulose to form LCCs [48,52,53], and this has been shown to greatly affect the digestibility of lignocellulosic biomass [54,55].

**Figure 6:**
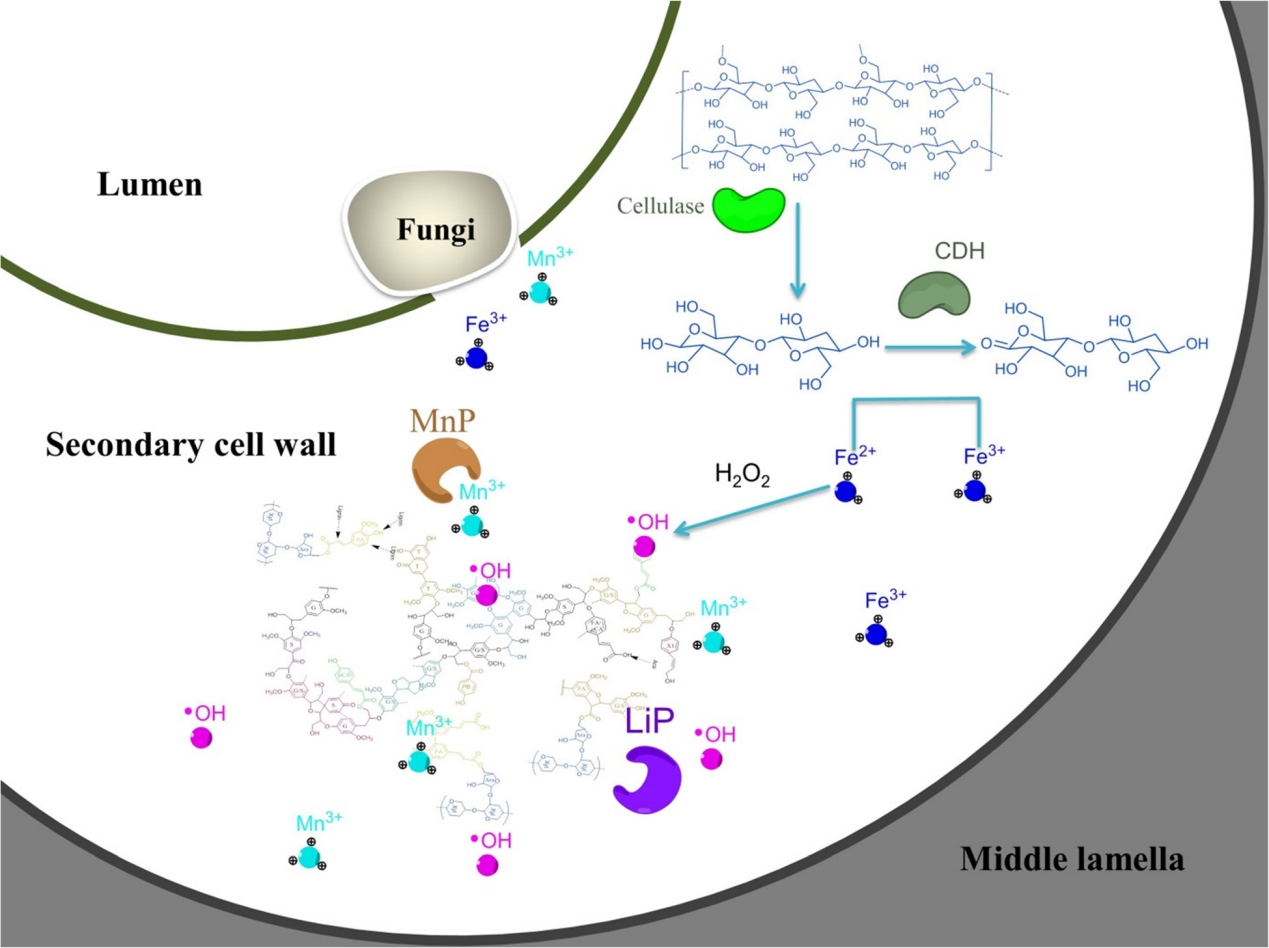
**A proposed scheme of wheat straw cell wall degraded by** ***Phanerochaete chrysosporium*** **.** Initial stage of the fungal growth is accompanied by hyphal attachment to the inner cell wall of the biomass. During growth, multiple enzymes are secreted, including cellulase, hemicellulase, and ligninase. Extracellular secretion of oxidases could participate in the generation of hydrogen peroxide and small carbon acids (such as acetate, succinate, and oxalate). Hydroxyl radicals, H_2_O_2_, and Fe^3+^ can be generated through synergistic interaction between cellobiose dehydrodrogenases (CDH), oxidases (copper radical oxidase among others), and the Fenton reaction. At the same time, Mn^2+^ is oxidized to Mn^3+^ by manganese peroxidase (MnP). The diffusion of these small radicals occurs predominantly in the secondary cell wall, which contains mainly the structural carbohydrates and aromatic backbone. The radicalization within the secondary cell wall contributes structural swelling by breaking the highly reactive ether linkages. This facilitates access of higher molecular weight enzymes.

However, there is still a question of how ligninases secreted by *P. chrysosporium* can selectively access these regions, since the compact structure of the cell wall rigidly prevents enzyme accessibility. Our previous proteome analysis of *P. chrysosporium*-spent wheat straw indicates the early secretion of lipases, oxidases, and esterases along with LiP that may enter the cell wall and break the lignin barrier [40]. During solid-state fermentation, fungal strains with detectable LiP activity show an increase in saccharification [56], which is enhanced with additional nutrients [45]. In fact, white rot fungi initially penetrate the lignocellulosic cell wall from the lumen to the secondary cell wall, followed by the middle lamella and cell corner [57,58]. In the fungal lignin degradation system, the theory of abiotic reaction on lignin degradation by lower molecular weight reagents, such as Fe^3+^, OH^•^, Mn^3+^, and H_2_O_2_, has recently resolved the puzzle of diffusion enzymes in the cell wall [59,60]. Up-regulation of cellobiose dehydrogenases (CDH), LiP, and MnP has been detected when *P. chrysosporium* is grown on a mixture of cellulose and lignin [29] and various types of lignocellulosic biomass [28]. CDH forms a hypha/cellulose complex [61] that acts on cellobiose and converts it into cellobionolactone by either the electron sink model or the electron chain model [60]. During this process, Fe^3+^ is reduced to Fe^2+^ that spontaneously involves the Fenton reaction to produce free OH^•^ radicals in the presence of H_2_O_2_, and further manipulate demethoxylation and hydroxylation of lignin [62] as an intermediary for Mn^3+^ attack [22]. The diffusion of these small free radicals can occur predominantly in the secondary cell wall, which mainly contains structural carbohydrates and the aromatic backbone. Radicalization within the secondary cell wall contributes to structural swelling by breaking highly reactive linkages (related to hydroxycinnamates and S units). This creates easy access for higher molecular weight enzymes. The scheme of the *P. chrysosporium* degradation pattern on the cell wall is shown in Figure 6. It is assumed that the biodegraded lignin relatively lacks specific phenolic units, thereby enhancing enzymatic hydrolysis. However, further research is needed on these processes.

## Conclusion

Biomass processing technologies and genetic engineering of plants can mitigate challenges in biomass conversion. Biodegradation of wheat straw by *P. chrysosporium* in terms of cellulose hydrolysis may not require complete depolymerization or deconstruction of the lignin complex. Rather, it may require dismantling specific substructures, including S units, hydroxycinnamates, γ-ending structures, and B and F units. Therefore, this study provides important clues for enhancing saccharification of lignocellulosic biomass and guiding genetic modification targets in lignin biosynthesis. This study also offers a new insight into the challenges in removing lignin from biomass during pretreatment and reducing its effects on saccharification and downstream processes. The results reveal that targeting lignin in the secondary cell wall promises to provide a sustainable and effective approach for biomass saccharification.

## Methods

### Preparation of *P. chrysosporium* and pretreatment

*P. chrysosporium* ATCC 24725 was maintained in potato dextrose agar (PDA). Cultivation occurred at 28°C in 250-mL flasks for the spores’ growth. After seven days of cultivation, fresh spores were suspended in 100 mL of sterilized water and subjected to count cell concentration with a Fisher Scientific hemacytometer. Wheat straw (*Triticum sativum)* grown in Moscow, Idaho was kept at 50°C until no weight loss was observed. One hundred grams of heat-dried wheat straw was soaked in 4 L of distilled water, autoclaved at 121°C for 20 minutes, and then the rest of the water was removed. After the wheat straw was cooled to room temperature, a 300-mL spore solution with 10^7^ cell/mL was inoculated at the surface, and then manually rotated for 10 minutes. Incubations were performed at 37°C for two, four, six, and eight weeks. Sterilized wheat straw samples (100 g) were treated in plastic containers inside a low temperature 815 incubator (Precision Scientific) (see Figure 1A). The containers were covered with eight-layer gauze pads in order to allow air exchange. Every 48 to 72 hours, 50 mL of water was evenly sprayed on the surface of the wheat straw while manually rotating the samples. At the end of the incubation periods, the samples were sequentially washed with water, dried, weighed, and chopped to size 0.25 mm (<60 mesh) before ball milling. The samples were then ball milled for 4 hours to a fine powder in a Retsch planetary ball mill, PM 100, with a zirconium dioxide ball at 300 rpm. The vessel rotated clockwise and anticlockwise every 3 minutes, with a 1-minute interval break. The experiments were performed in triplicate.

### Enzymatic hydrolysis

Enzymatic hydrolysis of the treated wheat straw was performed at 2% solids loading at 50°C for 48 hours under a cellulolytic hydrolysis solution. The cellulolytic solution was prepared by mixing 30 filter paper units (FPU)/g biomass cellulase from *Trichoderma reesei* ATCC 26921 (Sigma), 60 cellubiose units (CBU)/g biomass β-glucosidase Novozym 188 (Sigma), and 0.3% w/w hemicellulase from *Aspergillus niger* in a 50 mM sodium citrate buffer (pH 5.0). The sugar from the enzymatic hydrolysis was quantified with ion chromatography using an ion exchange chromatography apparatus (Dionex ICS-3000 DC IC) equipped with an electrochemical detector. All samples were separated using a Dionex Pac PA20 (3 mm × 150 mm) with a CarboPac PA20 Guard (3 mm × 30 mm). The flow rate was 0.5 mL/min, and the column temperature was maintained at 30°C. The samples were eluted isocratically with 20% 52 mM NaOH and 80% water. The column was flushed between samples with 100% 20 mM NaOH, followed by deionized water. The sugar yield was calculated by comparing released sugars to total sugar in the biomass. The experiments were performed in triplicate.

### Chemical composition analysis

Wheat straw samples were ground with a Wiley mill to pass through a 60 mesh screen and Soxhlet extracted with toluene:ethanol (2:1). The extractive free materials were further characterized by the two-stage acid hydrolysis method described by Standard Biomass Analytical Procedures (NREL) to determine the lignin and carbohydrate content [63]. The experiments were performed in triplicate.

### Solid-state ^13^C NMR analysis

Finely ground samples were packed in a 5.0 mm rotor with 1% w/w 3-(trimethylsilyl) propionic-2,2,3,4-*d*_*4*_ acid (TSP), and ^13^C CP-MAS NMR spectra were recorded at an ambient temperature in a Bruker DMX 400 spectrometer (NMR center, Washington State University). The approximate composition analysis (lignin and carbohydrate contents) followed Gilardi’s method (1995) [64]. The crystallinity of the biomass was estimated by dividing the area of the C4 crystalline peak (93 to 88 ppm) by the total area of the C4 peak (93 the 82 ppm) [65]. The experiments were performed in duplicate.

### Quantitative NMR analysis

Milled straw lignin (MSL) and cellulase enzyme lignin (CEL) were extracted from untreated and biological degraded wheat straw, respectively. Details on the isolation and quantitation procedure are described in our previous work [36]. The well-ground samples were extracted with ethanol:toluene (1:2) for 12 hours, followed by repeated washing with ethanol and water to remove extractives. The crude MSL was then isolated by dioxane/water (96:4, v/v) and yielded by evaporation of the solvent. After dissolving in acetic acid/water (9:1, v/v), the MSL was precipitated in excessive water, freeze dried, redissolved in dichloroethane/ethanol (2:1, v/v), and precipitated in ether again. The MSL removed residue was then subjected to enzymatic hydrolysis at 2% solids loading with 60 FPU/g cellulase and 120 CBU/g glucosidase in a 50 mM sodium citrate buffer (pH 5.0) at 50°C for 72 hours. Crude CEL was consequently isolated in a dioxane/water solution (96:4, v/v) and purified by the same purification procedure described above. The spectra of the isolated lignins were recorded in DMSO-d_6_ at 300 K on a Varian Inova 500 MHz spectrometer (Agilent Technologies, Santa Clara, CA, USA) operating at 499.86 MHz for ^1^H and 125.7 MHz for ^13^C. The residual solvent signal at 2.49 ppm for protons and 39.5 ppm for carbon was used for internal referencing of chemical shifts. The value represented the number of specific units in 100 aromatic rings.

### Cellulase adsorption to MSL and CEL

Cellulase adsorption to lignin was measured in a 15-mL tube containing 2% (w/v) lignin and 5 mL of 50 mM citrate buffer (pH 4.8) with various cellulose loadings (25 to 100 mg/g lignin). The samples were incubated at 4°C for 5 hours and were turned end-over-end manually every 10 minutes. After incubation, the tubes were centrifuged at 12,000 rpm for 10 minutes. The cellulase concentration in the supernatant was measured using a BCA kit (Sigma). The experiments were performed in duplicate.

### Calculation of adsorption parameters

The adsorption parameters (maximum adsorption capacity σ and equilibrium constant K) were calculated according to previously reported methods [7] using the Langmuir equation:

1 $$ \left[\mathrm{L}\mathrm{E}\right]=\frac{\upsigma \left[\mathrm{L}\right]\left[\mathrm{E}\mathrm{f}\right]}{\mathrm{K} + \left[\mathrm{E}\mathrm{f}\right]}d $$

where [LE] is the amount of adsorbed enzyme in mg/mL, [Ef] is the free enzyme concentration in mg/mL, σ is the maximum adsorption capacity in mg/mg lignin, [L] is the lignin concentration in mg/mL, and K is the equilibrium constant in mg of enzyme/mL. The affinity constant (A = 1/K) and binding strength (S = A × σ) were estimated.

## Additional files

Additional file 1: Table S1. Chemical composition (%) and weight (g) of wheat straw after biological degradation. Error bar represented as standard deviation. Additional file 2: Table S2. Klason lignin and carbohydrate content (%) of isolated MSL and CEL. Error bar represented as standard deviation. Additional file 3: Figure S1. The aromatic region of CEL isolated for two, four, six, and eight weeks treated and untreated wheat straw in HSQC spectra. H: *p*-hydroxyphenyl units; G: guaiacyl units; S: syringyl units; T: tricin units; *p*CA: *p*-coumarate; FA: ferulate; PB: hydroxybenzoate. Additional file 4: Figure S2. The aliphatic region of CEL isolated from two, four, six, and eight weeks treated and untreated wheat straw in HSQC spectra. A(α-OH): α-OH in β-O-4 linkage; A-H/G: coupled with H or G unit in *β*-O-4 linkage; A-S: coupled with S unit in β-O-4 linkage; A(γ-Ac): acylation by acetate in γ-OH; A(γ-pCA): acylation by pCA in γ-OH; Aγ: γ-OH in β-O-4 linkage; B: phenylcoumaran (β-5); C: pino/resinol (β-β); D: dibenzodioxocins (5-5′/4-O-β'); F: α,β-diaryl ethers; X1 (γ-OH): cinnamyl alcohol.

## Abbreviations

Aγ: γ-OH in *β*-O-4 linkage
A(γ-Ac): acylation by acetate in γ-OH
A(α-OH): α-OH in *β*-O-4 linkage
A(γ-*p*CA): acylation by *p*CA in γ-OH
A-H/G: coupled with H or G unit in *β*-O-4 linkage
A-S: coupled with S unit in *β*-O-4 linkage
B: phenylcoumaran (*β*-5)
C: pino/resinol (*β*-β)
CDH: cellobiose dehydrogenases
CEL: cellulase enzyme lignin
D: dibenzodioxocins (5-5′/4-O-*β*')
E: Spirodienone (*β-*1)
F: *α,β*-diaryl ethers
FA: ferulate
G: guaiacyl units
H: *p*-hydroxyphenyl units
MSL: milled straw lignin
PB: hydroxybenzoate
*p*CA: *p*-coumarate
S: syringyl units
T: tricin units
X1: cinnamyl alcohol
X2: cinnamyl aldehyde
