# Supplementary material for: Effects of lignin modification on wheat straw cell wall deconstruction by Phanerochaete chrysosporium
Source: Biotechnol Biofuels. 2014 Nov 29;7:161. doi: 10.1186/s13068-014-0161-3 (PMC4266972; doi:10.1186/s13068-014-0161-3)
Supplement: Additional file 1: Table S1. — Chemical composition (%) and weight (g) of wheat straw after biological degradation. Error bar represented as standard deviation. [file 13068_2014_161_MOESM1_ESM.docx]

Table S1. Chemical composition (%) and weight (g) of wheat straw after biological degradation. Error bar represented as standard deviation.

|  | **Control** | **two weeks** | **Four weeks** | **Six week** | **Eight week** |
| --- | --- | --- | --- | --- | --- |
| Arabinose | 2.75±0.12 | 0.49±0.01 | 0.61±0.06 | 0.63±0.04 | 0.48±0.06 |
| Galactose | 0.62±0.01 | 0.64±0.01 | 0.57±0.05 | 0.55±0.5 | 0.70±0.06 |
| Glucose | 52.46±1.08 | 51.23±0.55 | 47.14±0.48 | 48.99±0.34 | 47.13±0.55 |
| Xyl/man | 17.95±0.59 | 18.14±0.15 | 16.99±0.03 | 17.12±0.33 | 16.09±0.33 |
| Lignin | 18.3±0.56 | 20.0±0.57 | 22.55±1.20 | 26.2±1.41 | 27.35±1.63 |
| weight | 100 | 75.96±1.67 | 67.32±0.25 | 41.59±4.26 | 32.86±4.04 |
