# Supplementary material for: Effects of lignin modification on wheat straw cell wall deconstruction by Phanerochaete chrysosporium
Source: Biotechnol Biofuels. 2014 Nov 29;7:161. doi: 10.1186/s13068-014-0161-3 (PMC4266972; doi:10.1186/s13068-014-0161-3)
Supplement: Additional file 2: Table S2. — Klason lignin and carbohydrate content (%) of isolated MSL and CEL. Error bar represented as standard deviation. [file 13068_2014_161_MOESM2_ESM.docx]

Table S2.Klason lignin and carbohydrate content (%) of isolated MSL and CEL. Error bar represented as standard deviation.

|  | Arabinose | Xyl/man | Glucose | Klason lignin |
| --- | --- | --- | --- | --- |
| Control (MSL) | 0 | 0.16±0.01 | 0.15±0.01 | 98.22±0.14 |
| Control (CEL) | 0.58±0.04 | 1.40±0.02 | 0.23±0.02 | 87.16±0.02 |
| Eight weeks (MSL) | 0 | 0.28±0.01 | 0.29±0.02 | 95.12±0.26 |
| Eight weeks (CEL) | 0.50±0.02 | 1.44±0.08 | 0.29±0.04 | 87.25±0.23 |
