# Supplementary material for: Effects of lignin modification on wheat straw cell wall deconstruction by Phanerochaete chrysosporium
Source: Biotechnol Biofuels. 2014 Nov 29;7:161. doi: 10.1186/s13068-014-0161-3 (PMC4266972; doi:10.1186/s13068-014-0161-3)
Supplement: Additional file 3: Figure S1. — The aromatic region of CEL isolated for two, four, six, and eight weeks treated and untreated wheat straw in HSQC spectra. H: p-hydroxyphenyl units; G: guaiacyl units; S: syringyl units; T: tricin units; pCA: p-coumarate; FA: ferulate; PB: hydroxybenzoate. [file 13068_2014_161_MOESM3_ESM.docx]

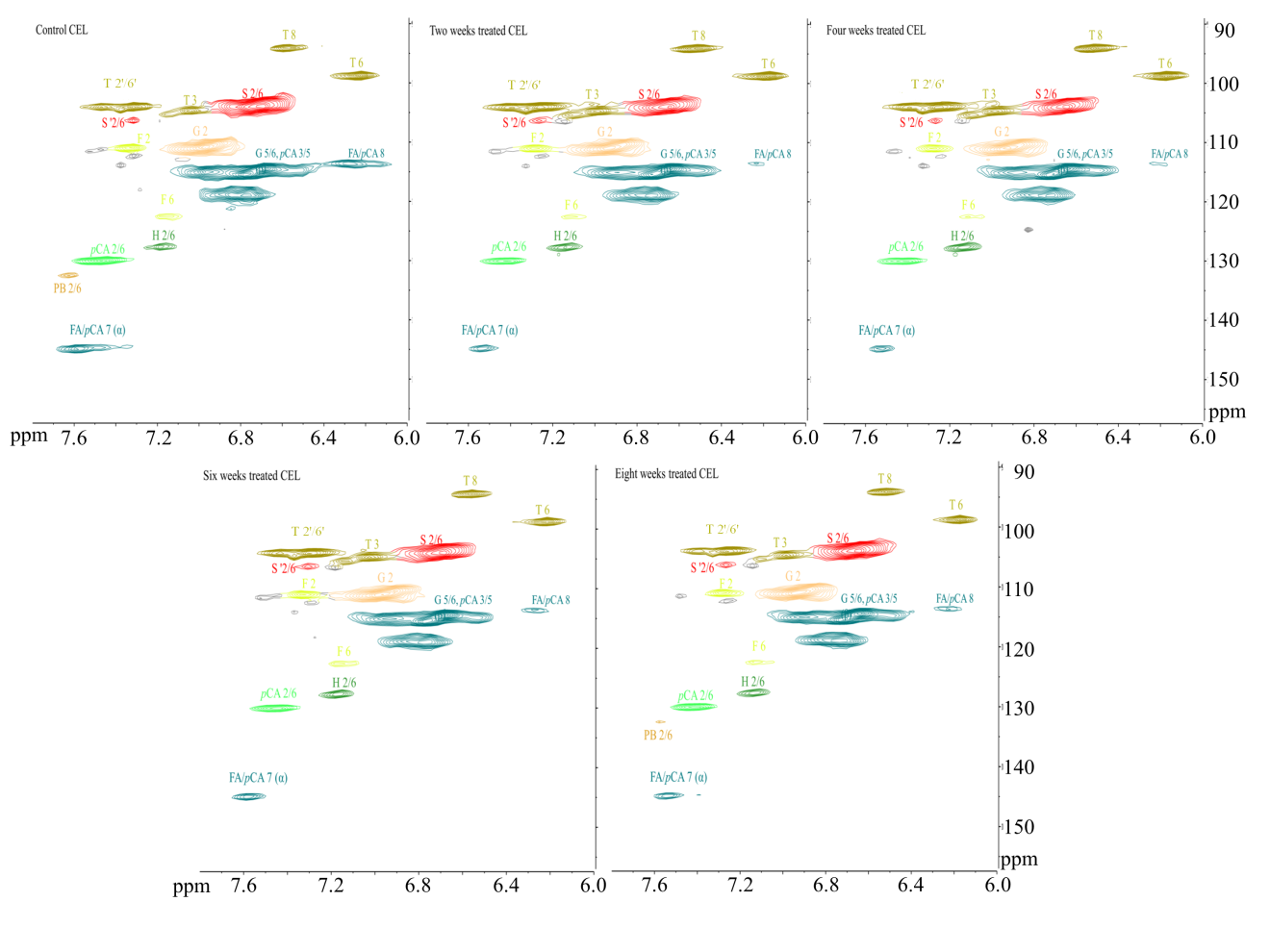


Fig. S1. The aromatic region of CEL isolated from two, four, six, eight weeks treated and untreated wheat straw in HSQC spectra. H: *p*-hydroxyphenyl units; G: guaiacyl units; S: syringyl units; T: tricin units; X1: cinnamyl alcohol; X2: cinnamyl aldehyde; *p*CA: *p*-coumarate; FA: ferulate; PB: hydroxybenzoate.
