# Supplementary material for: Effects of lignin modification on wheat straw cell wall deconstruction by Phanerochaete chrysosporium
Source: Biotechnol Biofuels. 2014 Nov 29;7:161. doi: 10.1186/s13068-014-0161-3 (PMC4266972; doi:10.1186/s13068-014-0161-3)
Supplement: Additional file 4: Figure S2. — The aliphatic region of CEL isolated from two, four, six, and eight weeks treated and untreated wheat straw in HSQC spectra. A(α-OH): α-OH in β-O-4 linkage; A-H/G: coupled with H or G unit in β-O-4 linkage; A-S: coupled with S unit in β-O-4 linkage; A(γ-Ac): acylation by acetate in γ-OH; A(γ-pCA): acylation by pCA in γ-OH; Aγ: γ-OH in β-O-4 linkage; B: phenylcoumaran (β-5); C: pino/resinol (β-β); D: dibenzodioxocins (5-5′/4-O-β'); F: α,β-diaryl ethers; X1 (γ-OH): cinnamyl alcohol. [file 13068_2014_161_MOESM4_ESM.docx]

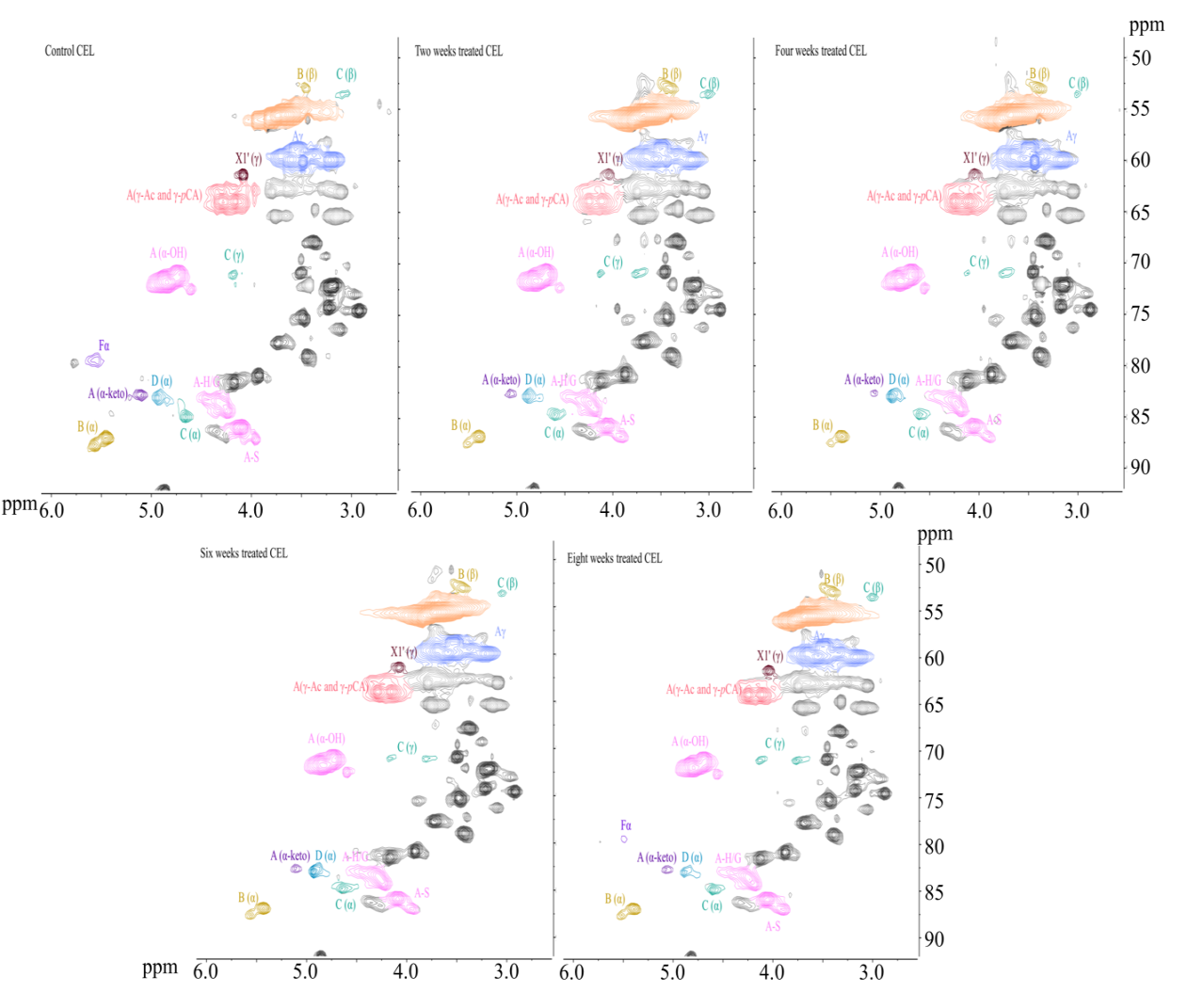


Figure S2. The aliphatic region of CEL isolated from two, four, six, eight weeks treated and untreated wheat straw in HSQC spectra. A(α-OH): α-OH in *β*-O-4 linkage; A-H/G: coupled with H or G unit in *β*-O-4 linkage; A-S: coupled with S unit in *β*-O-4 linkage; A(γ-Ac): acylation by acetate in γ-OH; A(γ-*p*CA): acylation by *p*CA in γ-OH; Aγ: γ-OH in *β*-O-4 linkage; B: phenylcoumaran (*β*-5); C: pino/resinol (*β*-β); D: dibenzodioxocins (5-5'/4-O-*β*'); F: *α,β*-diaryl ethers.
